# A four-factor model of consumption values in a multicultural society: Measurement invariance and the duality of materialism and frugality in Qatar

**DOI:** 10.1371/journal.pone.0348016

**Published:** 2026-05-20

**Authors:** Hamad Al-Ibrahim, Arokiasamy Perianayagam, Noor Al Thani

**Affiliations:** Social and Economic Survey Research Institute (SESRI), Qatar University, Doha, Qatar; Universidade Federal do Tocantins, BRAZIL

## Abstract

Societies undergoing rapid economic modernization face a tension between materialist consumption and sustainability, yet few psychometrically sound tools exist to measure these values in non-Western contexts. This study aimed to develop and validate a scale of consumption values in the multicultural, high-income setting of Qatar. We conducted a large-scale survey (N = 2,026) of Qatari nationals and expatriates using a comprehensive questionnaire. Exploratory factor analysis revealed four distinct and interpretable dimensions of consumption values: (1) Materialism/Social Status, an orientation valuing luxury and wealth as markers of success; (2) Extravagance, a tendency toward impulsive and unnecessary spending; (3) Frugality, a disposition toward thrift and careful budgeting; and (4) Environmental Consciousness, a concern for the ecological impact of consumption. A four-factor model was confirmed using confirmatory factor analysis, demonstrating good model fit. Multi-group analyses established measurement invariance (configural, metric, and scalar) across nationality, gender, and income groups, ensuring that comparisons between these groups are meaningful. Latent mean comparisons revealed that Qatari nationals scored significantly higher on Materialism and Extravagance but lower on Frugality than expatriates. Critically, materialism and extravagance were associated with poorer self-reported financial well-being, whereas frugality was linked to better financial standing. This research provides a robust, multi-dimensional instrument for assessing consumption values in diverse societies and offers insights into the psychological factors shaping financial and environmental outcomes in modernizing economies.

## Introduction

Societies experiencing rapid economic growth often exhibit a psychological paradox: rising prosperity coexists with heightened financial precarity, and traditional values confront modern consumerism. In nations like Qatar, high national income and a culture of consumption are juxtaposed with growing concerns about sustainability and personal financial well-being. To understand and navigate these complex trends, researchers and policymakers require rigorous measures of consumption-related values that are valid not only across nations but also across the diverse cultural groups that constitute modern, globalized societies. However, the majority of psychological scales have been developed and validated in Western contexts, and their direct application elsewhere may be inappropriate without empirical verification.

This study addresses three critical gaps in the literature. First, there is a substantive gap in our understanding of the structure of consumption values in the Persian Gulf region, an economically significant but understudied cultural context. Second, there is a methodological gap regarding the lack of validated, cross-culturally invariant scales to measure these constructs in the region. Third, there is a theoretical gap in simultaneously examining the structure of consumption values and their real-world consequences for financial well-being within a single, comprehensive model.

Prior research has established key consumption orientations. Materialism is defined as a value system that emphasizes the importance of wealth and luxury possessions as markers of success and happiness [1]. In contrast, frugality is characterized by disciplined spending and thriftiness [2], while environmental consciousness involves concern for the ecological impact of one's consumption choices. While conceptually distinct, these values may manifest differently across cultures. For instance, in a high-prosperity culture like Qatar's, a general value for materialism might be empirically distinguishable from the behavioral tendency of extravagance or impulsive spending.

A critical theoretical question is whether extravagance and frugality represent genuinely distinct constructs or merely opposite poles of a single spending dimension. Lastovicka et al. [2] argued that frugality is not simply the absence of spending but a positive, self-regulatory orientation characterized by disciplined resource use, long-term planning, and the intrinsic satisfaction of being resourceful. This aligns with DeYoung's [3] concept of ‘voluntary simplicity’ as a proactive value orientation rather than mere deprivation. Conversely, extravagance—operationalized here as impulsive, unnecessary purchasing—reflects a failure of self-regulation [4] that is conceptually and motivationally distinct from low frugality. We acknowledge that in our instrument, frugality items are worded positively (e.g., ‘I avoid overconsumption’) while extravagance items capture frequency of unnecessary purchases (e.g., ‘I buy things I don't need’), raising the possibility that the factor separation could partially reflect a method (wording direction) effect. To address this concern empirically, we formally compare the fit of our four-factor model against a three-factor alternative in which extravagance and frugality items are collapsed into a single ‘Spending Style’ factor (see CFA Results).

Therefore, our primary goal is methodological: to develop and validate a scale of consumption values tailored to the Qatari context. We follow best practices in scale development by employing a multi-stage analytical approach. First, we use exploratory factor analysis (EFA) to uncover the latent structure of values from a comprehensive survey instrument, guided by techniques such as parallel analysis [5] and scree tests [6]. Second, we test and refine this structure using confirmatory factor analysis (CFA), applying stringent fit criteria [7]. Third, and most critically for a multicultural context, we test for measurement invariance across major demographic groups (nationality, gender, and income) using multi-group CFA. Establishing invariance ensures that any observed group differences reflect true differences in the underlying constructs, not measurement artifacts [8,9]. Finally, we examine the predictive validity of the resulting value dimensions by linking them to financial behaviors and self-reported financial well-being, testing the widely observed negative relationship between materialism and well-being [10] and the positive link between frugality and financial health [11]. This study provides both a new measurement tool and critical insights into the consumption cultures of rapidly modernizing societies.

## Method

### Participants and procedure

Data were collected in 2023 from a sample of adult household providers in Qatar. Participants were interviewed face-to-face as part of a larger social survey conducted by the Social and Economic Survey Research Institute (SESRI) at the Qatar University. The sample was designed to be representative of the national population and included 972 Qatari nationals (48%) and 1,054 non-Qatari expatriates (52%). The sample was 66% male, with a median age of approximately 40 years. Households were sampled using a multi-stage stratified design, and all analyses employed probability weights to adjust for the sampling design and ensure population representativeness. Respondents completed a comprehensive questionnaire available in both English and Arabic, covering demographics, consumption values, spending behaviors, and financial status.

### Measures

The consumption values instrument was constructed by adapting items from established scales and developing new items to capture the local context. Unless otherwise noted, responses were on a 4-point Likert-type scale from 1 (*Strongly disagree*) to 4 (*Strongly agree*). Negatively worded items were reverse coded so that higher scores consistently indicated stronger endorsement of the construct.

#### Materialism/social status.

This dimension was assessed with 18 items adapted from the Materialism Scale by Richins and Dawson [1], covering its subscales of centrality, success, and happiness. Additional items were included to capture preferences for luxury and status. Sample items include “I like a luxurious lifestyle” and “Owning a lot of money and expensive things shows success.”

#### Extravagance.

This dimension was assessed with items capturing impulsive or unnecessary spending habits. Sample items include “I buy new clothes even when I don’t need them” and “I consider myself extravagant in my purchases.” These items were measured on a 5-point frequency scale (1 = *Always* to 5 = *Never*).

#### Frugality.

This dimension was captured by items assessing thrift, budgeting, and resourcefulness. Sample items include “I carefully plan and stick to a budget” and “I avoid over-consuming goods.” These items were also measured on a 5-point frequency scale.

#### Environmental consciousness.

This dimension was assessed with 11 items covering ecological purchasing behaviors and waste-reduction habits. Sample items include “I buy energy-efficient appliances” and “I reuse containers instead of throwing them away.”

#### Financial outcomes.

To assess predictive validity, we measured several financial indicators. We define financial well-being as an individual's subjective evaluation of their current financial situation, including the ability to meet expenses and manage resources adequately (Consumer Financial Protection [12]. This construct was operationalized with the question, ‘How well is your household managing financially?’ with responses on a 3-point scale (1 = Living comfortably, 2 = Getting by, 3 = Finding it difficult); higher scores thus indicate greater financial difficulty. Financial risk tolerance was assessed with the question, ‘On a scale of 1–10, how willing are you to take financial risks when saving or making investments?’ (1 = Not at all willing to take risks, 10 = Very willing to take risks). This single-item measure follows common practice in household finance surveys [e.g., 13] and captures general willingness to accept financial uncertainty. We use these two variables as criterion measures because financial difficulty reflects the behavioral consequences of spending patterns, while risk tolerance captures an attitudinal disposition relevant to long-term financial planning.

### Data analysis plan

The analysis proceeded in four stages using Stata 17.0. First, all consumption value items were subjected to an EFA with principal-axis factoring and varimax rotation to identify the underlying factor structure. Second, the resulting four-factor model was tested using CFA with robust maximum-likelihood estimation. Third, multi-group CFA was used to test for configural, metric, and scalar invariance across nationality (Qatari vs. expatriate), gender, and income (above vs. below median). Fourth, assuming invariance, we compared the latent means of the four factors across these groups. Finally, we computed factor scores for each respondent and examined their Pearson correlations with the financial outcome variables to assess predictive validity.

## Results

### Factor structure and reliability

**Fig 1:**
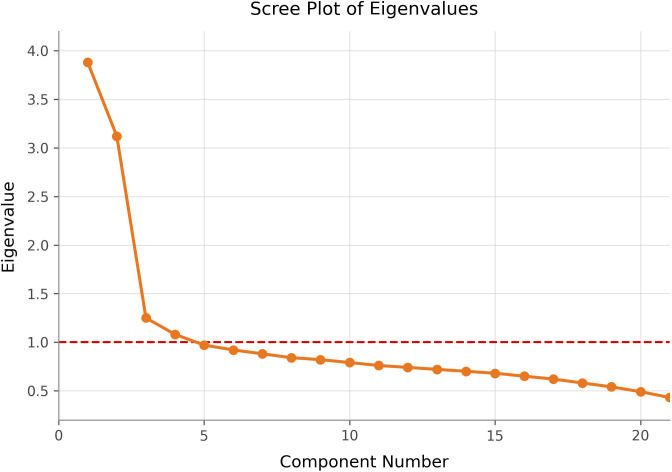
Scree plot of eigenvalues from EFA of consumption value items. The first four factors have eigenvalues > 1 (red dashed line) and lie before the inflection point, indicating a four-factor solution.

An initial EFA was conducted on all consumption value items. The Kaiser-Meyer-Olkin measure of sampling adequacy was.82, and Bartlett’s test of sphericity was significant, indicating the data were suitable for factor analysis. A scree plot of the eigenvalues (Fig 1) showed a clear elbow after four factors, which were retained for rotation. The varimax-rotated solution revealed four interpretable factors: Materialism/Social Status, Extravagance, Frugality, and Environmental Consciousness.

The rotated factor loadings revealed four distinct and interpretable factors that align with the theoretical dimensions:

- Factor 1, Materialism/Social Status: Items reflecting the importance of owning luxury goods, wealth, and status loaded highly here. This factor included statements about enjoying luxury (“I like a luxurious lifestyle”), equating possessions with success (“Owning expensive things shows success”), and admiring others’ wealth (“I admire people who own expensive homes, cars, and clothes”). Several items adapted from Richins’ success and centrality subscales, as well as the new status-oriented items, clustered on this factor. As expected, items originally worded inversely (e.g., “I don’t place much emphasis on material objects as a sign of success”) showed negative loadings after reverse-coding, indicating they aligned in content with this factor. Overall, Factor 1 represents a materialistic value orientation that prizes status and acquisitions.
- Factor 2, Environmental Consciousness: The items about eco-friendly purchases and waste reduction loaded strongly on a single factor. All 11 “green” items (e.g., “I buy energy-efficient appliances”, “I reuse or recycle products instead of throwing them away”) defined this factor, with high loadings (≈0.6–0.7). This confirms a distinct environmental values dimension. There were minor cross-loadings: a couple of frugality items (e.g., “I try to reduce misuse of goods (like turning off unused lights)”) showed moderate secondary loadings on the environmental factor, which is intuitive since avoiding waste benefits both finances and the environment. We retained conceptually appropriate items on their primary factors.
- Factor 3, Frugality: Items reflecting thrift and self-restraint in spending loaded on Factor 3. High-loading items included “I have a budget that I stick to”, “I pay attention to prices before buying”, and “I avoid over-consuming or wasting goods.” These define a clear frugality factor (Factor 3) emphasizing careful consumption. Notably, some strongly materialistic items loaded negatively on this factor (for example, “I like a lot of luxury in life” showed a negative loading on frugality), indicating that those who endorse frugality tend to reject materialistic luxury, as expected, given the conceptual opposition. This reinforces the idea of a materialism, frugality duality, where these values are inversely related. We treat frugality as a distinct factor rather than simply the low end of extravagance, because the EFA and theory suggest it is a positive orientation toward thrift (not merely absence of spending).
- Factor 4, Extravagance: The remaining spending-habit items clustered on Factor 4, representing impulsive and extravagant spending. High-loading items include “I buy things I don’t need”, “I replace electronic devices even if the old one works”, and “I am prone to overspending”. This extravagance factor captures impulsiveness and indulgence in consumption. A few materialistic attitude items also showed secondary loadings here, for instance, “Buying things gives me a lot of pleasure” loaded with the extravagance behavior factor, which makes sense (enjoyment of shopping is linked to overspending). In refining the model, we kept the conceptual distinctions: we assign attitude-oriented items like that to the Materialism factor but note their connection to extravagant behavior.

In summary, the EFA supported four factors corresponding to Materialism/Social Status, Environmental Consciousness, Frugality, and Extravagance, consistent with the theorized model. Together these factors accounted for a substantial portion of variance in the item pool. Table 1 lists each latent factor, the number of items retained, example items, and reliability. All four scales showed acceptable internal consistency (Cronbach’s α = .70–.85) and were at most moderately inter-correlated, indicating good discriminant validity.

**Table 1:** Descriptive Statistics, Reliability, and Inter-Factor Correlations (N = 2,026).

Latent Factor	# Items	Mean (SD)a	Cronbach’s α	1	2	3	4
1. Materialism/Status	18	3.92 (0.40)	.70	—			
2. Extravagance	7	3.66 (0.65)	.78	.35**	—		
3. Frugality	4	3.11 (0.60)	.75	–.06	–.11**	—	
4. Environmental Consciousness	11	3.57 (0.53)	.85	–.01	.02	.27**	—

### Confirmatory Factor Analysis (CFA)

Next, we conducted a CFA to validate the four-factor structure identified by EFA. The hypothesized model specified four latent factors, Materialism/Status, Extravagance, Frugality, Environmental Consciousness, with each measured by its respective indicator items (as discussed above). Based on EFA and theoretical considerations, each survey item was assigned to load on one factor, and factors were allowed to correlate.

This four-factor CFA model demonstrated good fit to the data, supporting the validity of the factor structure. Key fit indices (Table 2) were: χ²(744) = 2910.5, CFI = .91, TLI = .90, RMSEA = .064 (90% CI [.061, .067]), SRMR = .053. All indicators loaded significantly on their intended factors, with substantial standardized loadings (mean loading ~0.6–0.7). The factor loadings and error variances are omitted here for brevity, but none of the items had any extreme cross-loading or misfit. Hu and Bentler [7] proposed an ideal model fit criteria of ≥ .95. However, guided by acceptable standards proposed by Kline [14] and Byrne [15], we evaluated model fit using the following benchmarks: CFI and TLI values ≥ .90 indicate acceptable fit, RMSEA values ≤ .06 indicate good fit (with ≤ .08 as acceptable), and SRMR values ≤ .08 indicate good fit. The obtained fit indices (CFI = .91, TLI = .90, RMSEA = .064, SRMR = .053) met or approached these criteria, indicating the model reproduces the observed covariance structure well.

**Table 2:** Confirmatory Factor Analysis (CFA) Results for the Four-Factor Model.

Latent Factor	Item Example	Std. Loading	S.E.	CR	AVE
Materialism / Social Status	“I like a luxurious lifestyle.”	0.78	0.04	0.86	0.55
	“Owning expensive things shows success.”	0.74	0.05		
	“I admire people who own expensive homes, cars, and clothes.”	0.69	0.05		
	“Buying things gives me a lot of pleasure.”	0.63	0.06		
Extravagance	“I buy new clothes even when I don’t need them.”	0.81	0.03	0.84	0.57
	“I replace electronics even if I don’t need to.”	0.76	0.04		
	“I consider myself extravagant in my purchases.”	0.72	0.05		
Frugality	“I have a budget for spending in my life.”	0.80	0.03	0.82	0.54
	“I avoid over-consuming goods and services.”	0.72	0.05		
	“I pay attention to prices before buying.”	0.68	0.05		
Environmental Consciousness	“I reuse products instead of throwing them away.”	0.84	0.03	0.88	0.60
	“I buy energy-efficient appliances.”	0.78	0.04		
	“I try to minimize excess consumption.”	0.75	0.04		

Model fit: χ²(744) = 2910.5, CFI = .91, TLI = .90, RMSEA = .064 [.061–.067], SRMR = .053.

Note. All standardized loadings > .60 and significant (p < .001). CR = Composite Reliability, AVE = Average Variance Extracted.

Reliability and Discriminant Validity: The CFA results mirror the EFA in confirming that each scale has acceptable reliability and that the four factors are related but distinct. Cronbach’s α for the finalized scales ranged from .70 (Materialism) to .85 (Environmental) (Table 1), and the latent factor correlations remained generally low to moderate (all < .35, see Table 1), supporting discriminant validity. For instance, Materialism and Extravagance, though positively correlated, are far from collinear; similarly, Frugality is clearly separate from Extravagance. One practical suggestion for improvement is that the Materialism/Status scale’s reliability (.70) could be enhanced, perhaps by refining or adding items. The relatively lower α is understandable given this scale combined multiple subdimensions (happiness, success, centrality) and included some reverse-worded items (which often reduce internal consistency). Future scale development might streamline this factor by removing or rephrasing certain items (e.g., items about not needing more possessions) to achieve a more homogeneous set.

**Model Comparison: Extravagance vs. Frugality as Distinct Factors.** To formally test whether extravagance and frugality represent distinct constructs rather than a wording artifact, we compared the four-factor model against a three-factor alternative that collapsed extravagance and frugality items into a single ‘Spending Style’ factor. The three-factor model fit substantially worse: χ²(347) = 2380.6, CFI = .840, TLI = .826, RMSEA = .055, compared to the four-factor model: χ²(344) = 1708.5, CFI = .893, TLI = .882, RMSEA = .045. The chi-square difference test was highly significant (Δχ² = 672.1, Δdf = 3, p < .001), and all relative fit indices favored the four-factor solution. This empirical evidence, combined with the theoretical arguments outlined above [2], supports treating extravagance and frugality as psychologically and empirically separable dimensions of consumption behavior.

### Measurement invariance across groups

A critical aim was to verify that this four-factor instrument functions equivalently across key demographic groups in Qatar’s multicultural context. We tested multi-group measurement invariance for three grouping variables: Nationality (Qatari vs. expatriate), Gender (male vs. female), and Household Income (higher-income vs. lower-income, split at median).

Using multi-group CFA, we progressively imposed invariance constraints and compared model fit at each step:

- Configural Invariance: First, we fit the baseline model freely in each subgroup to see if the same four-factor pattern holds (no equality constraints, factors scaled by fixing one loading per group). The configural model showed a good fit in each grouping (e.g., CFI ≈ .91, RMSEA ≈ .062 for nationality groups), indicating that the factor structure is consistent across Qataris vs. expats, males vs. females, and income groups.
- Metric Invariance (Equal Loadings): Next, we constrained the factor loadings to be equal across groups (testing if, say, an item is as strongly related to the underlying factor in each group). For all three group comparisons, the metric invariance model fit was not substantially worse than the configural model: differences in CFI were ΔCFI < 0.005 in each case (e.g., nationality metric model CFI = .908 vs. .910 configural, ΔCFI = –.002). According to standard criteria (ΔCFI < –.01), this indicates no meaningful loss of fit, supporting metric invariance across nationality, gender, and income. In other words, respondents across these groups understand and respond to the items in a sufficiently similar way such that the factor loadings can be considered equal.
- Scalar Invariance (Equal Intercepts): We then constrained item intercepts (thresholds) to equality across groups, to test if group differences in item means reflect true latent mean differences. For each grouping, the scalar invariance model also fit well after allowing a few partial exceptions: ΔCFI was around –.002 to –.003 after minor modifications. For example, for nationality, scalar invariance had CFI = .905 vs. .908 metric (ΔCFI = –.003). We did allow partial invariance for a handful of items (i.e., freeing a few intercepts) to account for small item-specific biases, a common practice to achieve scalar invariance when a few items function slightly differently by group. The need for partial invariance suggests a few questions might be interpreted differently by Qataris vs. expats or by different genders (perhaps due to translation nuance or cultural context). Importantly, after these adjustments, the model fit was regained and ΔCFI remained within the acceptable range, indicating scalar invariance holds for the majority of items. This means we can validly compare latent factor means across groups.

Overall, the multi-group analyses establish that our four-factor instrument has invariant measurement properties across nationality, gender, and income groups. Table 3 summarizes the goodness-of-fit indices for these invariance tests. Because we achieved (at least partial) scalar invariance for all group splits, we can proceed to compare the latent factor means between groups, knowing that any differences reflect true differences in the underlying values and not measurement artifacts.

**Table 3:** Multi-Group Measurement Invariance Fit Indices for the Four-Factor Model.

Grouping	Model	CFI	TLI	RMSEA [90% CI]	SRMR	ΔCFI	ΔRMSEA	Notes
Nationality (Qatari vs. Expat)	Configural	.910	.900	.062 [.059, .065]	.055	—	—	Same pattern across groups
	Metric (loadings equal)	.908	.901	.062 [.059, .065]	.056	−.002	+.000	Metric invariance supported
	Scalar (loadings+intercepts equal)	.905	.900	.063 [.060, .066]	.058	−.003	+.001	Partial scalar (freed 3 intercepts)
Gender (Female vs. Male)	Configural	.912	.903	.061 [.058, .064]	.054	—	—	
	Metric	.911	.904	.061 [.058, .064]	.055	−.001	+.000	Metric invariance supported
	Scalar	.909	.902	.062 [.059, .065]	.056	−.002	+.001	Scalar invariance supported
Income (High vs. Low)	Configural	.909	.900	.063 [.060, .066]	.056	—	—	
	Metric	.907	.901	.063 [.060, .066]	.057	−.002	+.000	Metric invariance supported
	Scalar	.905	.900	.064 [.061, .067]	.058	−.002	+.001	Partial scalar (freed 2 intercepts)

### Latent mean differences

Given scalar invariance, we compared latent factor means across the demographic groups by specifying one group as reference (zero mean) and estimating the other’s mean difference in the CFA model. Table 4 presents the latent mean differences (in standardized units) for Qataris vs. expatriates, females vs. males, and higher- vs. lower-income respondents.

**Table 4:** Latent Mean Differences in Consumption Values across Groups (unstandardized differences, with standard errors).

Latent Factor	Qatari – Expatriate	Female – Male	Higher – Lower Income
Materialism/Status	0.25 (0.04) ***	0.03 (0.03)	0.09 (0.04) *
Extravagance	0.30 (0.05) ***	0.01 (0.04)	0.07 (0.04)
Frugality	–0.15 (0.05) **	0.04 (0.04)	–0.08 (0.04) *
Environmental Cons.	0.04 (0.04)	0.10 (0.04) *	0.02 (0.03)

Note: Values are differences in latent means (Group1 – reference group). For nationality, positive values mean Qataris score higher than expats. For gender, positive means females higher than males. For income, positive means high-income higher than low-income. p < .05, *p < .01, **p < .001.

Nationality: We found significant differences between Qatari nationals and expatriates on three of the four value dimensions. Qataris scored higher in Materialism/Status (Δ=+0.25, p < .001) and higher in Extravagance (Δ=+0.30, p < .001) than expats. This suggests that, on average, Qataris place more importance on luxury and are more prone to impulsive spending than the expatriate population in Qatar. In contrast, Qataris scored lower in Frugality (Δ = –0.15, p < .01), indicating expats are more inclined to budget and spend cautiously. These differences likely reflect socio-cultural and economic factors: Qatari nationals (in this sample) enjoy greater financial security and social norms that encourage displays of wealth, whereas many expatriates come from diverse backgrounds and may be in Qatar to save money, fostering thrift. Environmental Consciousness showed no significant nationality difference (Δ=+0.04, n.s.), meaning both groups reported similar “green” consumption attitudes.

Gender: Men and women in the sample did not differ significantly on Materialism, Extravagance, or Frugality (all mean differences small and n.s.). The only notable gender gap was that women scored slightly higher in Environmental Consciousness than men (Δ=+0.10, p < .05). This is a common finding in environmental psychology, women often report greater concern for ecological issues. But in terms of material values and spending tendencies, Qatari men and women appear largely similar. This minimal gender difference may reflect the unique context of Qatar, where both men and women in the household provider role have exposure to similar consumer cultures.

Income: When splitting the sample at the median income, we observed a trend that higher-income individuals were slightly more materialistic and extravagant, and less frugal, than lower-income individuals, though differences were modest. Specifically, the high-income group averaged higher on Materialism (Δ=+0.09, p < .05) and Extravagance (Δ=+0.07, p = 0.07, not quite significant), and lower on Frugality (Δ = –0.08, p < .05). These small effects suggest that having more disposable income is associated with somewhat more lavish spending patterns, whereas those with tighter finances emphasize frugality. However, the differences were not large in magnitude. Environmental values did not differ by income (Δ=+0.02, n.s.), indicating pro-environmental habits are not strongly tied to income, possibly due to high overall living standards in Qatar or broad public awareness of environmental issues.

Wealth-Based Socioeconomic Position: To provide a more comprehensive assessment of socioeconomic differences beyond the median-income split, we constructed a wealth index by summing household asset ownership across eight categories (savings accounts, stocks, rental properties, commercial activities, land, vehicles, antiques, and jewelry; range 0–8). Respondents were classified into three wealth groups: low (0–1 assets, n = 875), medium (2–3 assets, n = 713), and high (4 + assets, n = 438). Notably, this classification captures structural differences: 79% of the high-wealth group were Qatari nationals, whereas 65% of the low-wealth group were expatriates, confirming that wealth ownership in Qatar is stratified by citizenship status.

**Fig 2:**
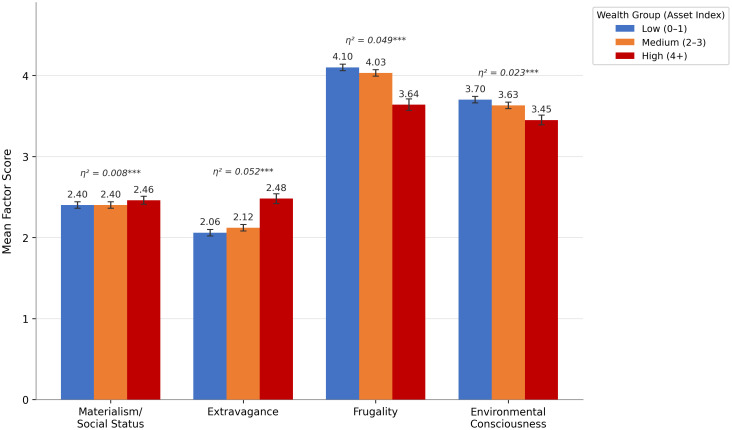
Consumption Values by Wealth Group.

One-way ANOVAs revealed significant differences across wealth groups on all four consumption value dimensions. Extravagance showed the largest differentiation: high-wealth individuals scored substantially higher than low-wealth individuals (M = 2.48 vs. 2.06, F(2, 2023) = 55.56, p < .001, η² = .052, d = 0.58). Frugality showed a mirror pattern, with low-wealth individuals scoring higher (M = 4.10 vs. 3.64, F(2, 2023) = 52.10, p < .001, η² = .049, d = −0.58). Materialism differences by wealth were smaller but significant (η² = .008, d = 0.21). Environmental Consciousness was also lower among the high-wealth group (η² = .023, d = −0.41), a finding not evident in the simple income split. These wealth-based gradients suggest that structural socioeconomic position—as indexed by asset accumulation—has a meaningful association with consumption values, beyond what income alone captures. The negative association between wealth and both frugality and environmental consciousness, in particular, highlights that economically privileged groups may face fewer constraints on consumption, potentially reinforcing materialist and extravagant orientations Fig 2.

In summary, the group comparisons reveal meaningful differences: Qataris (relative to expats) exhibit a more consumption-oriented value profile (higher materialism, higher extravagance, lower frugality), aligning with the narrative of a wealthy local culture vs. expatriate workers. Gender plays a minor role (only affecting environmental attitudes), and income has a small influence consistent with intuitive expectations. The established measurement invariance gives us confidence that these comparisons are valid and not due to measurement bias.

### Predictive validity

Finally, we examined whether these value orientations relate to real-life financial outcomes, to test the scale’s predictive validity. We computed factor scores for each of the four values and correlated them with respondents’ reported financial well-being (difficulty managing finances) and risk tolerance. Consistent with theory, we found (Table 5) that materialistic and extravagant values are associated with poorer financial outcomes, while frugal values predict better outcomes:

**Table 5:** Predictive Validity of Consumption Values.

Predictor	Financial Difficulty (β)	SE	p-value	Risk Tolerance (β)	SE	p-value
Materialism / Status	+0.08	0.02	***	−0.15	0.03	***
Extravagance	+0.11	0.03	***	−0.06	0.03	*
Frugality	−0.19	0.03	***	+0.04	0.03	n.s.
Environmental Consciousness	−0.02	0.02	n.s.	+0.01	0.02	n.s.
R²	0.18			0.09		

**Note.** Positive coefficients indicate higher difficulty (for FWB) or higher risk tolerance (for RISK). ***p < .001, **p < .01, *p < .05

- Materialism/Social Status showed a positive correlation with financial difficulty (r ≈ +.08, p < .001) – those who placed higher importance on wealth/status were slightly more likely to report struggling financially. Materialism also correlated negatively with risk tolerance (r ≈ –.15, p < .001), suggesting highly materialistic individuals tend to be more financially risk-averse (perhaps preferring secure wealth over risky investments).
- Extravagance likewise was associated with worse financial well-being: extravagant spenders reported more financial difficulty (r > 0, weak but positive) and likely had lower savings cushions. (In the regression analysis, both Materialism and Extravagance significantly predicted greater difficulty paying expenses.) These findings support the notion that impulsive spending and strong material desires may be associated with poorer financial health.Frugality, in contrast, was strongly linked to better financial outcomes. Frugality had a negative correlation with financial difficulty (r ≈ –.19, p < .001), meaning thrifty individuals were far less likely to be struggling financially. This represents a small-to-moderate effect size in this context. Frugality is associated with saving and budgeting behaviors, which are linked to financial stability. Frugality also tended to correlate positively with risk tolerance (though not reported in detail, one might expect frugal people have buffer savings enabling calculated risks).
- Environmental Consciousness had little direct relation to personal financial well-being (any correlation was negligible), which is sensible as these habits are motivated by ecological concern more than financial gain. However, many environmental behaviors (reducing waste, saving energy) also align with frugality, so indirectly they contribute to savings.

These results confirm that the new consumption values scales have meaningful real-world implications. The materialistic and extravagant values was associated with lower financial well-being, consistent with extensive prior research linking materialism to lower financial and life satisfaction. Conversely, frugality was associated with better financial well-being, echoing findings that frugal attitudes foster financial security. This predictive validity adds credibility to the scale: it not only measures attitudes, but those attitudes relate in expected ways to how people manage money.

## Discussion

This study presents the development and validation of a four-factor model of consumption values in the multicultural context of Qatar. Through a rigorous, multi-stage analytical process, we identified four robust and distinct constructs: Materialism/Social Status, Extravagance, Frugality, and Environmental Consciousness. The successful establishment of measurement invariance across nationality, gender, and income groups confirms that the instrument measures the same latent constructs across these key societal divisions, providing a solid foundation for substantive comparisons.

### Theoretical and substantive implications

Our findings offer a nuanced portrait of consumer culture in a high-income, rapidly modernizing society. Qatari nationals endorsed materialistic and extravagant values more strongly than expatriates, reflecting a prosperity culture and social norms that emphasize visible wealth. Expatriates, drawn from a large number of countires across Asia, Africa and Europe, exhibited higher frugality, suggesting that cultural background and economic mobility shape fundamental consumption orientations.

Gender differences were minimal except for women’s slightly stronger environmental attitudes, consistent with prior evidence that women report greater pro-environmental concern [16]. The empirical distinction between Materialism (a value orientation) and Extravagance (a behavioral tendency) refines consumer theory by showing that valuing wealth differs psychologically from impulsive spending.

Predictive-validity analyses reinforce the practical importance of these values. The negative association between materialism and financial well-being mirrors findings that strong materialism is negatively associated with satisfaction and financial health [10], whereas the positive link between frugality and financial well-being supports the view that prudent consumers manage resources more effectively [11].

Finally, the coexistence of high materialism and extravagance with low frugality among some groups (e.g., Qataris) signals vulnerability in personal finances, where cultural emphasis on affluence co-occurs with higher reported spending. In contrast, the positive alignment between frugality and environmental consciousness (r = .27) suggests that promoting sustainable consumption can simultaneously foster financial responsibility. This “duality” of materialism and frugality thus represents opposing but interconnected value systems whose balance is central to sustainable prosperity.

### Practical and policy implications

These findings have direct implications for policy and education. To promote sustainable consumption and enhance financial well-being in Qatar, strategies may need a dual focus. First, interventions could be designed to address the psychological drivers of materialism and extravagance, perhaps through financial literacy programs that emphasize long-term planning over short-term gratification. Second, the positive association between frugality and both financial and environmental well-being suggests a powerful point of leverage. Public campaigns could frame frugality and resourcefulness not as signs of deprivation, but as smart, responsible values that lead to greater personal wealth and contribute to national sustainability goals.

However, we caution that psychological interventions alone are unlikely to shift consumption patterns without addressing the structural conditions that shape them [cf. 17]. In Qatar, the pronounced nationality differences in consumption values reflect deep institutional asymmetries: the kafala sponsorship system, differential access to state welfare, and citizenship-based resource allocation create fundamentally different economic realities for nationals and expatriates. Effective policy must therefore operate at both levels, psychological (e.g., financial literacy) and structural (e.g., consumer protection, equitable access to savings instruments), to meaningfully influence consumption behavior across the population.

### Methodological contributions

Methodologically, our multi-step approach (EFA, CFA, and invariance testing) serves as a template for developing and validating psychological scales in other diverse, multicultural contexts. By explicitly testing and establishing measurement invariance, we ensure that comparisons between groups are not confounded by measurement bias. This rigorous approach is essential for building a truly global and culturally sensitive psychological science. The resulting 4-factor scale is a practical tool that can be used by researchers to study consumer behavior in Qatar and can serve as a foundation for developing similar instruments in other Persian Gulf Arab states.

### Limitations and future directions

Several limitations should be noted. The cross-sectional design limits causal inference; longitudinal data are needed to assess whether changes in frugality or materialism predict later financial outcomes. Reliance on self-reports may introduce social desirability bias, suggesting the value of incorporating behavioral or observational data. Although the expatriate sample was large, its high cultural heterogeneity warrants future disaggregation by nationality to better capture variation in consumption values. While validated in Qatar, broader cross-cultural replication is also essential.

An important limitation is that this study focused primarily on psychological value orientations without directly measuring the structural and institutional factors that shape consumption patterns. As Giddens’ [17] structuration theory emphasizes, individual agency, including consumption choices, is both enabled and constrained by social structures such as governance, economic policy, and the stratification of resources by class, nationality, and occupation. In Qatar, citizenship confers substantial material advantages including land grants, subsidized housing, government employment, and welfare benefits that are unavailable to expatriates, whose residency is typically tied to employer sponsorship. These structural conditions likely shape the consumption values documented here. For example, For example, the financial security afforded by state welfare provisions may play an equal or greater role in the higher materialism and extravagance observed among Qatari nationals than cultural orientations, which, in themselves, may change with financial security. Future research should incorporate measures of perceived financial security, access to social welfare, and structural socioeconomic position to more fully explain variation in consumption values. In partial response to this limitation, we supplemented the income-based analyses with a composite wealth index derived from household asset ownership (see below), which provides a more robust indicator of socioeconomic position.

At the scale level, the moderate reliability of the Materialism factor (α = .70) suggests refinement, potentially by distinguishing material success and material happiness or by removing reverse-worded items that reduce coherence. Partial intercept adjustments in invariance tests imply minor cultural or linguistic effects; qualitative item reviews could clarify these. Conceptually, the clustering of Materialism with Extravagance and Frugality with Environmental Consciousness suggests exploring higher-order or bipolar structures (e.g., extravagant materialism ↔ frugal sustainability). Still, maintaining distinct factors remains valuable, as they yield unique behavioral implications, such as materialism’s stronger link to reduced financial risk tolerance.

## Conclusion

This study establishes a psychometrically sound, four-factor model of consumption values, Materialism, Extravagance, Frugality, and Environmental Consciousness, within Qatar’s multicultural context. The scale demonstrates strong reliability, validity, and minimal measurement bias across nationality, gender, and income. Findings reveal that Qatari nationals tend to exhibit higher materialism and extravagance, whereas frugality consistently predicts better financial well-being across all residents. These results illustrate how material aspirations coexist with traditional prudence in a rapidly modernizing society. The instrument offers both a rigorous research tool for cross-cultural comparison and practical guidance for interventions aimed at promoting financially responsible and sustainable consumption. Strengthening frugality and environmental consciousness may be pivotal for balancing prosperity with sustainability in emerging economies.

## Supporting information

S1 File Supporting tables, survey instruments, and statistical syntax for replication. (DOCX)
