# Supplementary material for: A four-factor model of consumption values in a multicultural society: Measurement invariance and the duality of materialism and frugality in Qatar
Source: PLoS One. 2026 May 20;21(5):e0348016. doi: 10.1371/journal.pone.0348016 (PMC13189314; doi:10.1371/journal.pone.0348016)
Supplement: S1 File — (DOCX) [file pone.0348016.s001.docx]

**Supporting Information**

*A Four-Factor Model of Consumption Values in a Multicultural Society: Measurement Invariance and the Duality of Materialism and Frugality in Qatar*

Hamad Al-Ibrahim, Arokiasamy Perianayagam, Noor Al Thani

# S1 Table. Exploratory Factor Analysis: Rotated Factor Loadings (Varimax) and Communalities for Consumption Value Items

Note. Principal-axis factoring with varimax rotation (N = 2,026). Loadings ≥ |.40| shown in full; all loadings shown for selected items. h² = communality. * = reverse-coded item. Items shown are representative; full item set available from authors.

| **Item** | **Materialism** | **Extravagance** | **Frugality** | **Environment** | **h²** |
| --- | --- | --- | --- | --- | --- |
| "I like a luxurious lifestyle." | .77 | .18 | –.11 | .04 | .65 |
| "Owning expensive things shows success." | .73 | .20 | –.08 | .03 | .61 |
| "I admire people who own expensive homes, cars, and clothes." | .69 | .15 | –.05 | .01 | .52 |
| "Buying things gives me a lot of pleasure." | .63 | .22 | –.09 | .06 | .48 |
| "Expensive things impress me." | .68 | .17 | –.07 | .02 | .51 |
| "I don’t place much emphasis on material objects."* | –.55 | –.12 | .18 | .08 | .37 |
| "I buy new clothes even when I don’t need them." | .11 | .78 | –.12 | .05 | .67 |
| "I replace electronics even if I don’t need to." | .14 | .76 | –.10 | .03 | .62 |
| "I consider myself extravagant." | .20 | .72 | –.15 | .04 | .60 |
| "I buy things I don’t need." | .16 | .71 | –.14 | .02 | .57 |
| "I am prone to overspending." | .18 | .68 | –.13 | .01 | .53 |
| "I plan carefully before buying." | –.06 | –.18 | .72 | .21 | .62 |
| "I have a budget that I stick to." | –.08 | –.14 | .80 | .15 | .70 |
| "I avoid over-consuming goods." | –.04 | –.12 | .72 | .24 | .62 |
| "I pay attention to prices before buying." | –.03 | –.09 | .68 | .18 | .52 |
| "I reuse products instead of throwing away." | .02 | .06 | .19 | .79 | .68 |
| "I buy energy-efficient appliances." | .01 | .04 | .17 | .78 | .65 |
| "I try to minimize excess consumption." | –.02 | .03 | .22 | .75 | .63 |
| "I consider environmental impact before buying." | .03 | .01 | .15 | .73 | .57 |
| "I buy environmentally friendly products." | .04 | .05 | .14 | .71 | .55 |

# S2 Table. Confirmatory Factor Analysis: Standardized Factor Loadings, Composite Reliability, and Average Variance Extracted

Note. Model fit: χ²(744) = 2910.5, CFI = .91, TLI = .90, RMSEA = .064 [.061–.067], SRMR = .053. All standardized loadings > .60 and significant (p < .001). CR = Composite Reliability. AVE = Average Variance Extracted. α = Cronbach’s alpha.

| **Factor** | **Range of Std. Loadings** | **CR** | **AVE** | **α** |
| --- | --- | --- | --- | --- |
| Materialism / Social Status | .63 – .78 | .86 | .55 | .70 |
| Extravagance | .72 – .81 | .84 | .57 | .78 |
| Frugality | .68 – .80 | .82 | .54 | .75 |
| Environmental Consciousness | .75 – .84 | .88 | .60 | .85 |

# S3 Table. Multi-Group Measurement Invariance Fit Indices Across Nationality, Gender, and Income

Note. Invariance criterion: |ΔCFI| ≤ .010 (Chen, 2007; Cheung & Rensvold, 2002). Partial scalar invariance for nationality (3 intercepts freed) and income (2 intercepts freed).

| **Grouping** | **Model** | **CFI** | **TLI** | **RMSEA [90% CI]** | **SRMR** | **ΔCFI** | **ΔRMSEA** | **Decision** |
| --- | --- | --- | --- | --- | --- | --- | --- | --- |
| Nationality | Configural | .910 | .900 | .062 [.059,.065] | .055 | — | — | Accept |
|  | Metric | .908 | .901 | .062 [.059,.065] | .056 | –.002 | +.000 | Accept |
|  | Scalar (partial) | .905 | .900 | .063 [.060,.066] | .058 | –.003 | +.001 | Accept |
| Gender | Configural | .912 | .903 | .061 [.058,.064] | .054 | — | — | Accept |
|  | Metric | .911 | .904 | .061 [.058,.064] | .055 | –.001 | +.000 | Accept |
|  | Scalar | .909 | .902 | .062 [.059,.065] | .056 | –.002 | +.001 | Accept |
| Income | Configural | .909 | .900 | .063 [.060,.066] | .056 | — | — | Accept |
|  | Metric | .907 | .901 | .063 [.060,.066] | .057 | –.002 | +.000 | Accept |
|  | Scalar (partial) | .905 | .900 | .064 [.061,.067] | .058 | –.002 | +.001 | Accept |

# S4 Table. Latent Mean Differences (Unstandardized) in Consumption Values Across Nationality, Gender, and Income Groups

Note. Values are differences in latent means (Group 1 – reference). Standard errors in parentheses. *p < .05, **p < .01, ***p < .001.

| **Factor** | **Qatari – Expatriate** | **Female – Male** | **High – Low Income** |
| --- | --- | --- | --- |
| Materialism / Status | +0.25 (0.04)*** | +0.03 (0.03) | +0.09 (0.04)* |
| Extravagance | +0.30 (0.05)*** | +0.01 (0.04) | +0.07 (0.04) |
| Frugality | –0.15 (0.05)** | +0.04 (0.04) | –0.08 (0.04)* |
| Environmental Consciousness | +0.04 (0.04) | +0.10 (0.04)* | +0.02 (0.03) |

# S5 Table. Predictive Validity: Regression of Financial Outcomes on Consumption Value Factors

Note. Model 1 = consumption value factors only. Model 2 = with structural controls (nationality, gender, age, wealth index). Standardized β coefficients reported. Robust standard errors (HC1). n.s. = not significant.

| **Predictor** | **Fin. Diff. (β)** | **SE** | **p** | **Risk Tol. (β)** | **SE** | **p** | **Model** |
| --- | --- | --- | --- | --- | --- | --- | --- |
| Materialism/Status | +0.08 | 0.02 | <.001 | –0.15 | 0.03 | <.001 | 1 |
| Extravagance | +0.11 | 0.03 | <.001 | –0.06 | 0.03 | .04 | 1 |
| Frugality | –0.19 | 0.03 | <.001 | +0.04 | 0.03 | n.s. | 1 |
| Env. Consciousness | –0.02 | 0.02 | n.s. | +0.01 | 0.02 | n.s. | 1 |
| R² | 0.18 |  |  | 0.09 |  |  | 1 |
| Materialism/Status | +0.06 | 0.02 | <.01 | –0.08 | 0.03 | <.01 | 2 |
| Extravagance | +0.12 | 0.03 | <.001 | –0.11 | 0.03 | <.001 | 2 |
| Frugality | –0.23 | 0.03 | <.001 | +0.11 | 0.03 | <.001 | 2 |
| Env. Consciousness | –0.01 | 0.02 | n.s. | +0.02 | 0.02 | n.s. | 2 |
| Qatari (nationality) | –0.06 | 0.03 | <.05 | –0.15 | 0.03 | <.001 | 2 |
| Female (gender) | +0.08 | 0.02 | <.001 | –0.04 | 0.03 | n.s. | 2 |
| Wealth index | –0.16 | 0.03 | <.001 | +0.18 | 0.03 | <.001 | 2 |
| Age | –0.03 | 0.02 | n.s. | –0.06 | 0.02 | <.05 | 2 |
| R² | 0.143 |  |  | 0.090 |  |  | 2 |

# S6 Table. One-Way ANOVA of Consumption Values by Wealth Group (Asset-Based Socioeconomic Index)

Note. Wealth groups based on composite asset index (sum of 9 binary asset ownership indicators). Means with standard deviations in parentheses. Low: 0–1 assets (n = 875); Medium: 2–3 assets (n = 713); High: 4+ assets (n = 438). η² = partial eta-squared. *p < .05, **p < .01, ***p < .001.

| **Factor** | **Low (0–1 assets)** | **Medium (2–3)** | **High (4+)** | **F (df)** | **η²** |
| --- | --- | --- | --- | --- | --- |
| Materialism/Status | 2.40 (0.38) | 2.40 (0.39) | 2.46 (0.42) | 4.3* | .008 |
| Extravagance | 2.06 (0.65) | 2.12 (0.63) | 2.48 (0.68) | 55.6*** | .052 |
| Frugality | 4.10 (0.56) | 4.03 (0.58) | 3.64 (0.64) | 52.1*** | .049 |
| Env. Consciousness | 3.70 (0.51) | 3.63 (0.52) | 3.45 (0.55) | 23.7*** | .023 |

# Appendix A – Survey Instruments (English and Arabic)

**English Version:** The questionnaire began with demographics, then Section 1 asked agreement (1 = Strongly disagree to 4 = Strongly agree) with statements about consumption values (e.g., “I like a luxurious lifestyle,” “Expensive things impress me,” “Owning a lot of money and expensive things shows success”). Section 2 covered status-seeking goods (e.g., “Buying luxury products makes people notice me”). Section 3 included lifestyle questions: 5-point frequency items on habitual spending (“I replace electronic devices with new ones even if I don’t need them,” “I buy clothes I don’t need,” “I stick to a budget,” “I avoid overusing goods,” etc.) and environmental practices (“I buy energy-efficient appliances,” “I reuse containers instead of throwing away,” “I minimize wasteful consumption,” etc.). The final sections assessed risk attitudes (1–10 scales) and financial well-being (e.g., “How well is your household managing financially?”).

**Arabic Version:** The Arabic questionnaire mirrored the English wording with cultural adaptation, covering the same topics and scales. A bilingual translation is provided in the full instrument document.

***Sample Items (English):***

**Materialism:** “I like a luxurious lifestyle.” “Expensive things impress me.” “I admire people who own expensive homes, cars, and clothes.”

**Extravagance/Frugality:** “I buy new clothes even though I do not need them.” “I try hard to reduce misuse of goods and services.” “I pay attention to the price of food I order.” “I have a budget for spending.” “I consider myself extravagant in my purchases.”

**Environmental Consciousness:** “I consider the environmental impact before making purchases.” “I buy environmentally friendly products.” “I minimize wasteful consumption for future generations.” “I reuse products such as shopping bags, containers, and glass instead of throwing them away.”

# Appendix B – Statistical Syntax for Replication and Verification

The following Stata 17 code illustrates the analytic steps used to replicate and validate the four-factor model of consumption values. Equivalent R lavaan syntax is available upon request.

***Code Snippet 1. Data Setup and Weighting***

use "CP_weighted.dta", clear

svyset [pweight = household_weight]

***Code Snippet 2. Exploratory Factor Analysis (EFA)***

factor material1-material18 consneg1-consneg11 envibe1-envibe6 waste1-waste5, pcf mineigen(1)

screeplot, factors(10)

factor material1-material18 consneg1-consneg11 envibe1-envibe6 waste1-waste5, factors(4)

rotate, varimax

estat loadings, cutoff(.40)

***Code Snippet 3. Confirmatory Factor Analysis (CFA)***

sem (Materialism -> material1-material18@1) ///

(Extravagance -> consneg1-consneg7@1) ///

(Frugality -> consneg8-consneg11@1) ///

(Environment -> envibe1-envibe6 waste1-waste5@1), standardized

estat gof, stats(all)

***Code Snippet 4. Measurement Invariance (Multi-Group CFA)***

sem ..., group(nationality) ginvariant(none)

sem ..., group(nationality) ginvariant(mcoef)

sem ..., group(nationality) ginvariant(mcoef mcons)

estat ginvariant

***Code Snippet 5. Predictive Validity Models***

predict mat_score extrav_score frug_score env_score if e(sample), latent

regress financial_difficulty mat_score extrav_score frug_score env_score, robust

regress risk_tolerance mat_score extrav_score frug_score env_score, robust

***Code Snippet 6. Wealth Index and Extended Models***

* Create wealth index from assets

egen wealth_index = rowtotal(assests1-assests9), missing

xtile wealth_q = wealth_index, nq(5)

* ANOVA by wealth group

oneway mat_score wealth_q, tabulate

oneway extrav_score wealth_q, tabulate

* Extended regressions with structural controls

regress financial_difficulty mat_score extrav_score frug_score env_score ///

i.is_qatari i.female wealth_index age, robust
